# Social and executive functioning in individuals with autism spectrum disorder without intellectual disability: The case–control study protocol of the CNeSA study

**DOI:** 10.3389/frcha.2023.1149244

**Published:** 2023-04-21

**Authors:** Federica Donno, Carla Balia, Jessica Boi, Mirko Manchia, Alessandro Zuddas, Sara Carucci

**Affiliations:** ^1^ Section of Neuroscience & Clinical Pharmacology, Department of Biomedical Science, University of Cagliari, Cagliari, Italy; ^2^ Child & Adolescent Neuropsychiatric Unit “A. Cao” Paediatric Hospital, ASL Cagliari, Cagliari, Italy; ^3^ Unit of Clinical Psychiatry, University Hospital Agency of Cagliari, Cagliari, Italy; ^4^ Unit of Psychiatry, Department of Medical Science and Public Health, University of Cagliari, Cagliari, Italy; ^5^ Department of Pharmacology, Dalhousie University, Halifax, NS, Canada

**Keywords:** autism spectrum disorder, neuropsychological functioning, autonomic functioning, control design, social cognition, hot executive functions, cold executive functions, study protocol

## Abstract

Several studies suggest that children and adolescents with autism spectrum disorder (ASD) often present deficits in executive functions (EFs). The research on cold EF shows a high heterogeneity across different cohorts of patients as well as different study designs, while studies investigating hot EF and their relationship with different ASD phenotypes are still limited and related only to specific domains, although this concept could contribute to clarify the phenotypical variability by explaining the difficulties encountered by individuals with ASD in daily life, where stimuli are often emotionally charged. With the aim to identify specific neuropsychological profiles in children and adolescents with ASD without intellectual disability, we designed a study protocol comparing a clinical sample of individuals with ASD to aged-matched (10–17 years) typically developing controls (TDC) on a neuropsychological test battery investigating both “cold” and “hot” EF with the purpose of further investigating their relationships with ASD symptoms. Autonomic measures including heart rate, heart rate variability, skin conductance, and salivary cortisol were also recorded before/during/after the neuropsychological testing session. This paper describes the case–control study protocol named “*Caratterizzazione NEuropsicologica del disturbo dello Spettro Autistico, senza Disabilità Intellettiva, CNeSA study*,” its rationale, the specific outcome measures, and their implications for the clinical management of individuals with ASD and a precision medicine approach.

## Introduction

Autism spectrum disorder (ASD) is a complex, lifelong, and multifactorial neurodevelopmental condition with onset in the first years of life. The Diagnostic and Statistical Manual of Mental Disorders, Fifth Edition (DSM 5) (1) introduced the term “spectrum” to highlight the broad heterogeneity of etiologies, onsets, clinical entities, and prognostic trajectories. Furthermore, individuals affected by ASD present heterogeneous neuropsychological profiles, due to the combination of several genetic and environmental factors that complexify the diagnosis and tailored interventions (2).

Children and adolescents with ASD without intellectual disability (ID) can use compensatory strategies for dealing with their difficulties in social contexts thus improving their social functioning (3, 4). However, they maintain significant difficulties in grasping social cues and other intentions, and have limited intuitive judgment skills and more inflexible decision-making processes (5, 6) as well as a lower sensitivity to rewards rather than punishments (7). Furthermore, individuals with ASD without ID may present atypical emotional responses to a stimulus and high aversive motivation toward social stimuli (8) with consequent impairment in their social functioning during daily life. Even when they present correct moral judgment, individuals with ASD tend to estimate certain transgressions or unfair acts more seriously than those with typical development, showing they rely on more rigid moral criteria (9).

Several theories tried to explain the core of social functioning difficulties in individuals with ASD (10–12). The hypothesis of executive disfunction (13, 14) includes deficits in the following functions: attention; flexibility and set-shifting; planning; inhibitory control; generativity; and working memory (15). Early studies reported a relationship between set-shifting impairment and restricted and repetitive behaviors (16, 17) and difficulties in facing changing situations (14). Disorders in selective attention were associated with restricted and repetitive behaviors (18, 19) while deficits on working memory were associated with difficulties in social communication and interaction, as the ability to take others perspective, social reciprocity and initiation (20). Moreover, a lack of generativity could compromise the quality of communication, due to the difficulty in generating ideas relevant to the context of conversation with others (21). Despite these results, the research on executive dysfunction in ASD shows contrasting results for the high variability in study designs and cohorts of patients (22, 23).

Furthermore, an early impairment in multisensory and sensory–motor integration (24, 25) is widely observed in children with ASD.

In children with ASD, the severity of sensory symptoms and intelligence quotient (IQ) seem to predict motor abilities, suggesting a relationship between the sensory, motor, and cognitive domains (26). A severe complex sensory processing impairment also appears to impact their motor variability (27).

In a seminal study, children with ASD aged younger than 4 years performed fewer exploratory actions on objects within a naturalistic setting, or engaged in repetitive, primitive sensorimotor actions compared to their typically developing (TD) peers who instead were more prone to explore action possibilities during means-end tasks (28). Difficulties in both fine and gross motor abilities can limit infants’ active exploration of objects and their ability to discover means-end relationships (29, 30).

The sensorimotor difficulties are often associated with behavioral motor problems, such as stereotyped behavior and restricted interests. Other studies found the presence of a relationship between early motor delay and later communication delay in infants at risk for ASD (31, 32), with a cascading effect on all aspects of neurodevelopment, including detail perception, motor planning, social communication, and cognitive domains.

In the last decade, a distinction between “cool” and “hot” executive functions (EFs) (33) has been introduced with the aim to discriminate the purely cognitive processes from those elicited by affective stimuli or related to emotionally salient situations in which emotional and cognitive processes are integrated to generate a behavior (34, 35). The “hot” EFs could contribute to explain the difficulties encountered by individuals with ASD in daily life where stimuli are often charged emotionally.

The high difficulty in managing daily changes, unpleasant events, negative social interactions, and external sensory stimuli also leads individuals with ASD to experience greater stress than neurotypical individuals (36).

Several studies suggest that heart rate (HR), heart rate variability (HRV), electrodermal activity (EDA), and cortisol levels appear to be altered in the ASD population: children with ASD seem to present a chronic state of hyperarousal (37–39), a psychophysiological inflexibility to stimuli (e.g., appropriate vagal withdrawal to attention-demanding stimuli) (40), and greater physiological responses to threat (41). Although individuals with ASD present a greater lower overall regulation than their typically developing peers, the ASD patients without intellectual impairment demonstrate more autonomic flexibility and responsiveness to stimuli (42) than those with intellectually impaired ASD (39). Moreover, ASD in individuals without intellectual impairment exhibits more autonomic responsivity showing more variable cardiac responding to familiar vs unfamiliar social situations in comparison to TD controls (TDCs) who do not show changes across stimuli (42).

Other studies investigating stress levels by measuring the salivary cortisol also report higher cortisol levels in individuals with ASD compared to both younger autistic individuals and age-compared TDCs during social interactions with peers (43, 44).

Numerous studies report the presence of a relationship between autonomic nervous system (ANS) activity and social and communication functioning in individuals with ASD (45–48). Relative to TDCs, patients with ASD present greater electrodermal activity during feedback rewards and the greatest increases were more likely exhibited by children with more repetitive symptoms, reduced executive functions, and internalizing symptoms (46).

According to these notions, some studies support the hypothesis that stress, either acute or chronic, affects cognitive performances and the way in which individuals with ASD perceive, understand, and react to the social world (49, 50). Excessive levels of stress have been associated with poorer performance in short-term memory tasks, learning, and attention tasks (51, 52). It is not yet clear, however, whether the cognitive dysfunctions observed in individuals with ASD entails a greater susceptibility to stressful situations or, conversely, whether stress produces an influence on the performance of cognitive tasks.

Moreover, to date, the studies investigating both hot and cold EFs are still limited, and the research is often focused on singular domains.

Considering the current literature, further research is required to understand the neuropsychological characteristics of patients with ASD in both domains of hot and cold EFs and how they differ from TDCs. Exploring the relation between symptom phenotypes, neuropsychological functioning and autonomic nervous system response will enhance the current knowledge on the neurobiological mechanisms underlying the difficulties presented by individuals with ASD. This may allow the identification of cognitive and physiological pathways underlying the disorders and improve the intervention strategies to support patients' autonomy in daily life activities.

Furthermore, the assessment of physiological parameters in association with the behavioral performance could help the clinician to understand the way the individuals with ASD process the experiences related to hot and cold tasks.

With this purpose in mind, we designed the present protocol to compare a clinical sample of individuals with ASD to aged-matched TDCs to investigate their neuropsychological functioning and the relationships with ASD symptoms.

## Methods and analysis

### Study design

This is a monocentric study including two phases: a screening and clinical assessment visit (phase I); and a case–control study (phase II) comparing the neuropsychological and autonomic functioning profiles of children and adolescents with ASD without ID to age-matched TDCs.

### Participants

Two cohorts of children and adolescents (aged 10–17 years and 10 months at screening visit) of both genders were enrolled in the study.

Group 1 (ASD group) included children and adolescents with a clinical diagnosis of ASD according to DSM 5 criteria without intellectual disability (IQ score ≥80).

Group 2 (TDC group) included typically developing children and adolescents without any psychopathology, matched to the ASD group for age, gender, and IQ.

### Eligibility criteria

#### Inclusion criteria for all participants

For eligibility, participants from both groups had to be aged 10–17 years and 10 months at the screening visit with an IQ >80 measured with Wechsler IQ scales (Wechsler Intelligence Scale for Children—Fourth Edition, WISC-IV or Wechsler Adult Intelligence Scale-IV, WAIS-IV) administered within 2 years before enrollment into the study.

#### ASD group inclusion criteria

Participants in the ASD group had to comply with the following requirements:

• Diagnosis of ASD in accordance with the DSM 5 criteria formulated by a qualified clinician according to normal clinical practice;
• Drug-naïve for psychotropic medications or off any psychotropic medication [psychostimulants, antipsychotics, serotonin and norepinephrine reuptake inhibitors (SNRIs), mood stabilizers, or antidepressants] within the last 6 months before the screening visit;
• Signed informed consent and absent documents provided by the individual's parent/legal guardians and the patients;
• Participants meeting the criteria for co-morbid Attention Deficit and Hyperactivity Disorders (ADHD), Anxiety, or Post Traumatic Stress Disorder (PTSD) as well as language and motor disorders were not excluded from the study (as for the clinical judgment of the investigator).

#### TDC group inclusion criteria

The participants included in the TDC group had to comply with the following requirements:

• A total score on the Social Communication Questionnaire (SCQ) below the clinical range for ASD: ≤10;
• Drug-naïve for psychotropic medications;
• Signed informed consent and absent documents provided by the individual's parent/legal guardians and the TDCs.

#### Exclusion criteria ASD group

Potential eligible participants for the ASD group were excluded from participation in the study if they met the following criteria:

• IQ <80 (Wechsler IQ scales, within the last 2 years before enrollment);
• Presence of a primary DSM 5 diagnosis of schizophrenia-related disorders, schizophrenia, bipolar disorder, depression;
• Presence of any acute or unstable medical condition compromising the reliability of the study;
• The participant had any psychotropic medication (psychostimulants, antipsychotics, SNRIs, mood stabilizers, or antidepressants) within the last 6 months before the beginning of the study;
• Biological siblings of the participants were already included within the ASD group.

#### Exclusion criteria TDC group

Potential TDCs were not enrolled into the study if they met any of the following criteria:

• IQ <80 (Wechsler IQ scales, within the last 2 years before enrollment);
• The participant was treated with any psychotropic medication (psychostimulants, antipsychotics, SNRIs, mood stabilizers, or antidepressants);
• The presence of a primary DSM 5 diagnosis of ADHD, oppositional defiant disorder (ODD), conduct disorder (CD), or any other psychiatric condition;
• The presence of any acute or unstable medical condition compromising the reliability of the study.

### Sample size calculation

To compare the performance between the ASD and TDC groups, a sample size of 40 ASD cases and 40 TDCs had a statistical power of >80% at the 0.05 level to detect group differences with a moderate effect size allowing for the inclusion of covariates (sex, site, children vs adolescents, IQ, co-morbidity with ADHD) (53).

### Enrollment

The ASD group was composed of inpatients or outpatients or clinical referrals from community centers and their parents were informed about the study by the clinical team or the study investigators. TDCs were identified through other clinical departments or from ASD participants’ family members or classmates who wanted to participate in the study.

Before starting any procedures, the parents/legal guardian and the child/adolescent provided written informed consent and the assent for participating in the study.

All participants were free to withdraw from the study at any time, for any reason, and without any consequences to their clinical treatment. The investigator could decide to withdraw an individual from the study for urgent medical or psychiatric reasons and a new participant was recruited from the same group (ASD/TDC) and gender as the individual who withdrew from the study. Data collected until the point of withdrawal were collected and included in the final analysis.

### Study procedures

**Figure 1:**
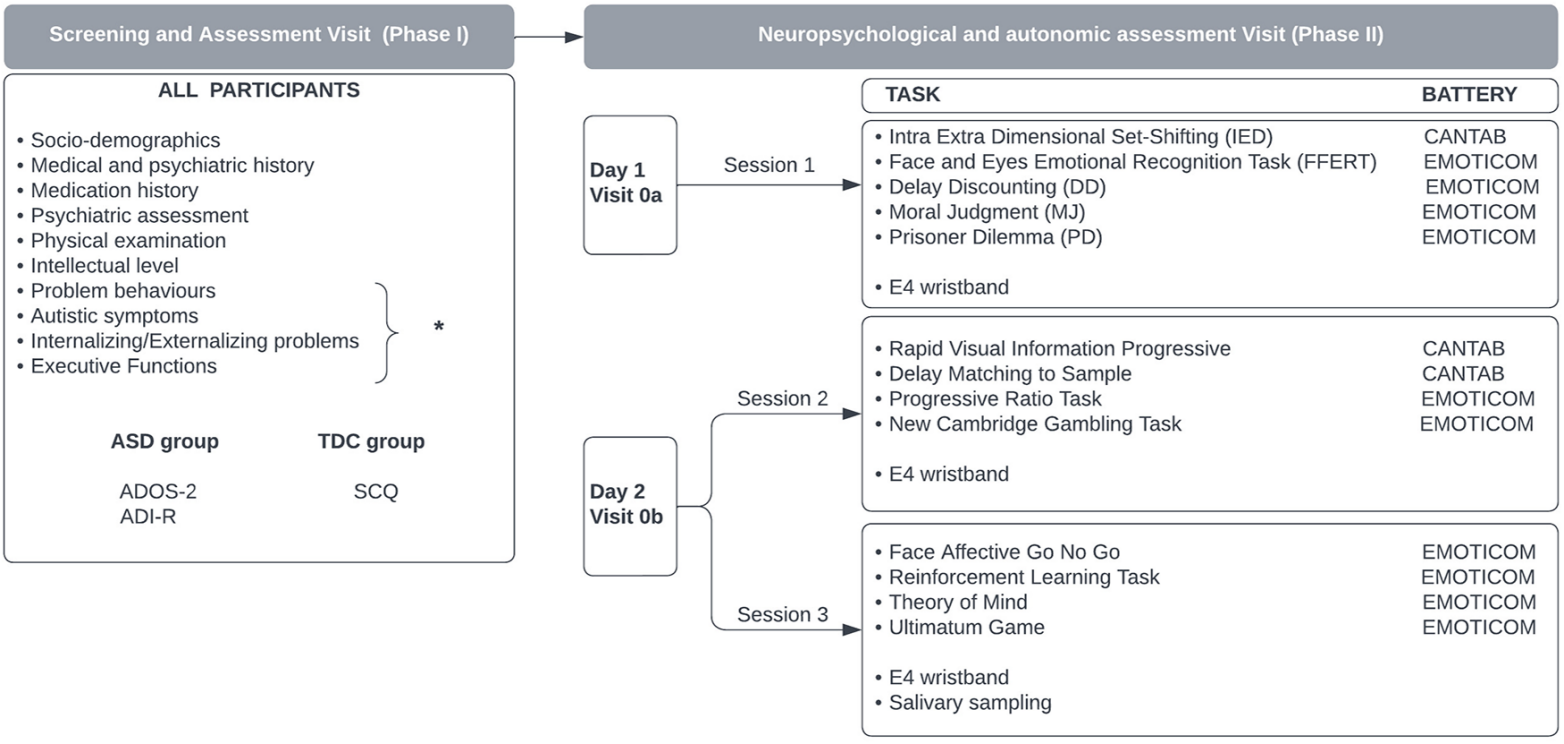
Study design. *The intellectual level is measured using Weschler scales (WISC-IV or WAIS-IV); behavioral problems are assessed with Kiddie-SADS-PL, CPRS-RS, NCBRF-TIQ, MOAS, ICU, C-GAS, and CGI; the assessment of autistic symptoms includes ADOS-2, SCQ, SRS-2, and EQ-40; internalizing/externalizing symptoms are evaluated with CBCL, TRF, and YSR; the assessment of executive functions includes the BRIEF questionnaire.

This study is a non-interventional case–control study divided into two phases: the screening and clinical assessment visit (phase I); and the case–control study including neuropsychological testing and physiological measures collection (phase II). To reduce the fatigue effect on testing performance, the case–control phase was divided into 2 days (visits 0a and 0b) (Figure 1; see also Supplementary Material Table S1: study design).

#### Screening and clinical assessment visit (phase I)

After receiving the signed informed consent and assent, sociodemographic information and data on medical, psychiatric, and pharmacological history of participants as well as medical history of relatives were collected for all participants and all criteria for enrollment were verified.

The screening session included the following (see Supplementary Material Table S2: clinical assessment; SM1: Description of screening and clinical instruments):

• WISC-IV (54) or WAIS-IV (55);
• Kiddie Schedule for Affective Disorders and Schizophrenia—Present and Lifetime Version (Kiddie-Sads-PL) (56).

An evaluation of autistic symptoms was different among the two groups.

Children/adolescents with ASD underwent a detailed psychiatric assessment for autistic symptoms using the following principal instruments for the diagnosis of ASD:

• Autism Diagnostic Interview—Revised (ADI-R) (57);
• Autism Observational Scale—Second Edition (ADOS-2) (58).

Parents of the participants included in TDC group completed the SCQ (59). A score of 10 is used as the cutoff for excluding autism in the participant.

Further assessments included the following:

• Modified Overt Aggression Scale (MOAS) (60);
• The Nisonger Child Behaviour Rating Form (NCBR-TIQ) parent version (61);
• Clinical Global Impression-Severity (CGI-S) (62);
• Children's Global Assessment Scales (C-GAS) (63).

**Participant forms:**

• Self Report (YSR) (64);

**Parent and teacher forms:**

• Social Responsiveness Scale—Second Edition (SRS-2) (65);
• Child Behavior Checklist (CBCL), Teacher Report Form (TRF) (64);
• Conner's Parent Rating Scale-Revised (CPRS-RS) (66);
• Behavior Rating Inventory of Executive Function (BRIEF) (67);
• Empathy Quotient (EQ-40) (68);
• Inventory of Callous Unemotional Traits (ICU) (69).

#### Vital signs, body temperature, height, and weight

Vital signs (blood pressure, heart rate), body temperature, height, and weight were recorded; a physical examination was also performed.

#### Case–control study (phase II)

Within 1 month from the screening visit, each participant took part in the case–control study consisting of neuropsychological testing (Figure 1). The computerized test battery was split into three sessions lasting 3 hours in total to perform over 2 days (visits 0a and 0b) at a maximum interval of 1 week.

In the first of the 2 days (visit 0a), the first session of neuropsychological tests comprising five tasks was administered lasting approximately 50 min in total (Table 1); during the second day (visit 0b), the participants completed the second and third sessions, each consisting of four tasks (Table 2). The second and third sessions were separated by an interval of 45 min during which the participant rested.

**Table 1:** First neuropsychological assessment session (visit 0a).

Neuropsychological tasks	Functions	Category
Intra/extra-dimensional set-shifting (IED)	Attentional set formation maintenance, shifting, and flexibility	Cold executive functions
Face and Eyes Emotion Recognition Task (FEERT)	Emotion recognition	Emotional processing
Delay Discounting (DD)	Impulsivity, rate of discount across delays, and probabilities	Impulsivity
Moral Judgment (MJ)	Moral emotions	Social cognition
Prisoner Dilemma (PD)	Social decision-making, cooperation	Social cognition

**Table 2:** Second neuropsychological assessment session (visit 0b).

Neuropsychological tasks	Functions	Category
Rapid Visual Processing (RVP)	Sustained attention	Cold executive functions
Delay Matching to Sample (DMS)	Visual matching and short-term visual recognition memory	Cold executive functions
Progressive Ratio Task (PRT)	Incentive motivation, motivational “breakpoint”	Motivation and reward
New Cambridge Gambling Task (NCGT)	Value-based decision-making, reward and punishment sensitivity, risk-taking behavior	Motivation and reward
Face Affective Go No Go Task (FAGNG)	Attentional affective bias	Emotional processing
Reinforcement Learning Task (RLT)	Learning based on reward and punishment	Motivation and reward
Ultimatum Game (UG)	Social decision-making, sensitivity to fairness and tendency to inflict punishment	Social cognition
Theory of Mind (ToM)	Social ToM information preference in ambiguous scenes	Social cognition

During all three sessions, the autonomic parameters HR, HRV, and EDA were collected through the application of the E4 wristband worn by the participant 20 minutes before the start until the end of the session.

During visit 0b, two samples of saliva were collected from all participants: 5 min before the first testing session and immediately after the end of the second session, in order to detect salivary cortisol levels.

#### The neuropsychological test battery

In this study, a computerized battery of neuropsychological tasks was used with the aim of exploring both the “cold” and “hot” EFs. All tasks were administered using a touchscreen tablet (10.1-inch screen) and included tasks from the neuropsychological test batteries “Cambridge Neuropsychological Automated Battery” (CANTAB; Cambridge Cognition: RRID:SCR_003001 https://www.cambridgecognition.com/cantab/) (70) and the EMOTICOM battery (71).

Three different domains of cold EFs (the sustained attention, the acquisition and maintenance of rules, and the set-shifting abilities and the visual working memory) were evaluated using three tasks from the CANTAB battery.

The tasks chosen to evaluate the domains of hot EFs (emotional processing, motivation and reward, impulsivity and social cognition, social decision-making) were derived by the EMOTICOM battery. For a detailed description of the administered neuropsychological tasks, see Supplementary Material: SM2: Neuropsychological Assessment.

A screening test was first administered with the aim of making the individual familiar with the tablet and other test materials. Within each visit, the presentation order of the tests was randomized for each participant.

#### Physiological measures

The stress levels due to task administration were evaluated through physiological measures collection (Table 3).

**Table 3:** Autonomic measures and instruments.

Category	Tools	Measures of stress response
Autonomic Measures	E4 wristband	Heart rate (HR) Heart rate variability (HRV) Skin conductance (EDA)
	Salivary sampling	Salivary cortisol (pre and post)

#### Autonomic measures using Empatica E4.

During the neuropsychological assessment, task-related stress and arousal levels were assessed with the application of the Empatica E4 wristband recording the autonomic measures HR and HRV by a photoplethysmography technique, and EDA. The bracelet was placed on the wrist of the non-dominant hand of the participant. The recording started at least 5 min before the testing session, while the participant was at rest, to yield baseline, and continued during the performance of the whole neuropsychological test battery.

During the test administration, a “tag event” function was used to delimit the beginning and the end of each test with the purpose of comparing stress and arousal levels during different neuropsychological tasks. The tag event delimiting the end of the task does not correspond with the start of the next task. Data collected during the period between the end of a task and the beginning of the next were excluded from the analysis.

#### Collection of saliva cortisol samples

During the second day of neuropsychological testing (visit 0b), two samples of saliva were collected with a “passive drool” method. Each 0b visit was scheduled in the morning to collect a baseline sample in a time window between 8:00 and 9:30, 5 min before the start of the first testing session. The stress sample was collected at the end of the second session, depending on the time needed for the execution of all tasks. The time of both collections was reported in the patient's case report form (CRF) to consider the circadian trend of cortisol by age.

If the participant had trouble spitting, sugar- and flavor-free chewing gum were provided to assist salivation. They were asked to rinse their mouth with water and then waited approximately 1 minute before saliva collection.

All samples were centrifuged after collection and then frozen and stored at −20°C until assay. Cortisol levels were extracted by an external lab (San Raffaele Hospital, Milano, Italy).

A difference between salivary cortisol levels at baseline and at the end of administration was calculated, within and between the two groups.

### Outcome measures

Primary outcome measures included the following: quantitative and qualitative behavioral measures evaluated through the neuropsychological test battery: measures of cold (sustained attention, attentional set formation maintenance, shifting, visual matching, and short-term visual recognition memory) and hot EFs (emotion recognition, attentional biased, reward-punishment sensitivity, moral judgment, cooperation, theory of mind, test completion, motivation). These were calculated using the following:

• response latency (reaction times);
• accuracy (number or proportion of errors).

The following physiological measures were recorded: heart rate; heart rate variability; skin conductance at rest and during the test performance; and salivary cortisol levels before and after testing session.

The secondary outcome measures were as follows: clinical measures to assess the association between autistic symptoms severity; co-morbidities; problem behaviors obtained by the administration of tests, interviews, rating scales, and questionnaires; and neuropsychological profile. These were obtained using the following:

• Screening questionnaires: SRS-2, CBCL, TRF, YSR, CPRS, BRIEF, ICU;
• Screening rating scales: MOAS and Nisonger;
• CGI-S, C-GAS.

### Handling and storage of data and documents

Data were handled confidentially and anonymously: a different participant identification code was used to link the data to each individual. The key to the code was safeguarded by the investigators. Demographic data (name, address, etc.) and identification numbers were coupled in a file, which was saved on a password-protected PC, only accessible to the investigators. The handling of personal data complied with Personal Data Protection Acts.

A unique code was given to each collected salivary sample, consisting of the type of sample, the visit session and the date of collection, and a unique, consecutive number. The code was not based on the patients’ initials and birthdate.

### Statistical analysis

The cognitive-behavioral measures for each task include mean reaction time, number or proportion of errors, and other quantitative measures.

The group differences will be evaluated using chi-square or one-way analysis of variance (ANOVA) tests in case of the data meeting the assumptions of normality and homogeneity of variance, while the effect of covariates will be explored using analysis of covariance (ANCOVA) and, thereafter, by determination of simple effects or interactions. Non-parametric tests (e.g., Mann–Whitney *U* test) or bootstrap-based non-parametric ANOVA will be used for variables that do not respect these assumptions. Simple and multiple and logistic regression models will be applied to the whole sample and to the ASD group. A mixed repeated-measures analysis will be used to compare the performance of ASD to the TD group within each task when appropriate.

Variables including age, gender, IQ, and co-morbidities such as ADHD, anxiety, and depression, will be also investigated as covariates.

Correlations, simple and multiple regressions, and ANCOVAs will be used to investigate the demographic, clinical, neuropsychological, and neurophysiological predictors to establish their role as moderating or modulating variables with symptom severity.

If, as expected, the raw cortisol values are positively skewed, they will be normalized using log transformation. To assess the group differences in cortisol variations, mixed repeated-measures ANOVAs will be performed with the group variable as a between-subjects factor and time (pre- and post-testing) as a within-subjects factor; the collection time of the samples will be used as a covariate.

One-way ANOVAs will be used for group comparisons of HR, HRV, and skin conductance means during the task performance. To quantify HR, HRV, and skin conductance responsiveness to the stress related to the performance during tasks, the change relative to baseline will be calculated. A clustering analysis will be also used to group individuals belonging to the whole sample in classes not defined a priori, according to autonomic characteristics.

## Discussion and clinical implications

ASD is a condition characterized by impaired socioemotional skills and patterns of restricted and repetitive interests and behaviors lasting in general for a lifetime. Although patients with ASD without ID implement compensatory strategies to face difficulties, most of them experience negative experiences, such as social isolation, and have few opportunities for sociability (72, 73). When they make friends like their TD peers, compared to the latter they experience a poorer quality of friendship (74). The presence in ASD individuals of hyper moral behaviors, such as scolding classmates who break the school rules or trying to socialize with peers or monopolizing speeches, make individuals with ASD vulnerable to bullying episodes. Furthermore, their difficulties in recognizing social cues [e.g., difficulties understanding the communicative intent of gaze (75, 76)], the tendency to incorrectly interpret others’ behavior (77, 78) as well as difficulties in interpreting verbal communication (79, 80) compromise the identification of bullying episodes, leading them to experience uncomfortable emotions.

To date, published studies on the neuropsychological functioning of ASD show contrasting results due to methods issued without providing exhaustive answers in explaining the neuropsychological dysfunctions in the ASD population. Studies on social decision-making as cooperation indicated lower correct predictions of others’ moves compared with TDCs (81) and an improvement of this ability with age (82, 83). Moreover, for individuals with ASD, their cooperation relies on more rigid criteria that does not differ depending on the morality of the interacting partner (84). In contrast, other studies report similar cooperation behavior in autistic and non-autistic individuals (85). Autistic individuals appear to make less use of contextual cues and reported less emotion reaction to the scenarios described in vignettes (86, 87).

In addition, the studies on reward-based decision-making using the gambling task reported contrasting results. Whereas the study by Faja et al. (46) showed a similar pattern of gambling selection between individuals with ASD and TDCs, the study by Yechiam et al. showed that, unlike TDCs, individuals with ASD presented fewer advantageous choices and difficulties in developing and maintaining a congruent choice strategy switching from a deck to another (88).

Considering the lack of knowledge in the field and the high heterogeneity of the ASD, the present study will help to better define the neuropsychological and autonomic characteristics underpinning ASD in order to provide useful information to identify the underlying potential neuropsychological/physiological mechanisms behind the clinical symptoms. Although several therapeutic strategies have already been developed to help autistic patients in dealing with social and emotional difficulties, including video modeling (89, 90), emotional recognition training (91, 92), social stories (93), and social skill training (94, 95), our study could contribute to develop further targeted intervention strategies.

Targeted interventions should consider the importance of factors that typically contribute to the valuable perception of social situations, including emotions and motivation, the stimuli relevance, the reward and punishment sensibility, the attentional affective bias vs. stimuli, and the uncertainty valence. For example, individuals with autism can orient their attention toward a stimulus rather than another leading to dysfunctional decision-making thus compromising their social functioning and their autonomy development. The results of this study will help to provide the basis for developing more effective psycho-educational strategies aimed at improving patients’ autonomy and at enhancing their social skills.

### Strengths and limitations of this study

The main strength of the present study protocol is to investigate, at the same time, both domains of hot and cold EFs as well as the autonomic functioning in a cohort of individuals with autism.

Another strength of this study is the wide neuropsychological and clinical characterization of the ASD sample within a narrow age range including different sources of information (parents, teachers, and the individuals themselves).

On the other hand, the co-morbidity with other neurodevelopmental disorders, such as ADHD or specific learning disorders, could represent a limitation of the study due to their influence on the task performances (e.g., tasks requiring reading words could be less suitable for patients with dyslexia, therefore requiring additional efforts; the length of tasks could influence the performance in children with hyperactivity or inattention symptoms). Moreover, the main limitation of this study is that the Emoticom tasks are not yet standardized in the pediatric population and the battery administered in this study is an “experimental” version.

## Ethics and dissemination

The present study was approved from the local Ethical Commitee (*Comitato Etico Indipendente*) of Cagliari University Hospital on 28 March 2018 and conducted in accordance with the principles of the Declaration of Helsinki. Before starting any study procedure, all participants' parents/legal guardians, patients, and controls signed an informed consent and assent document, respectively, as provided for by the national law. The results of the study will be disseminated through peer-reviewed publications and at scientific conferences.

## Conclusions

An integrative model of neuropsychological functioning in ASD would better explain the difficulties with ASD. An understanding of neuropsychological and autonomic functioning and the relation with ASD symptoms may lead to defining more effective intervention behavioral strategies that represent first-line therapy due to the poor responsivity to pharmacological treatments.

The assessment, which integrates both domains of cold and hot EFs, can provide insights into the executive deficits that hinder the development of the skills necessary in ecological social contexts.
