# Supplementary material for: Social and executive functioning in individuals with autism spectrum disorder without intellectual disability: The case–control study protocol of the CNeSA study
Source: Front Child Adolesc Psychiatry. 2023 Apr 21;2:1149244. doi: 10.3389/frcha.2023.1149244 (PMC11731623; doi:10.3389/frcha.2023.1149244)
Supplement: Supplementary file 1 [file Table1.docx]

**Supplementary Material Table (S1): study design**

|  | **Study schedule of events** | | | |
| --- | --- | --- | --- | --- |
|  | | **Phase I** | **Phase II** | |
| **Visit**  **Week** | | **Screening Visit**  **-4 to -1** | **Visit 0a**  **0** | **Visit 0b**  **0 - 1** |
| Informed Consent/Assent | | ✓ | ✓ |  |
| Inclusion/Exclusion Criteria | | ✓ | ✓ |  |
| Socio-demographics | | ✓ |  |  |
| Family medical history | | ✓ |  |  |
| Medical and psychiatric history | | ✓ |  |  |
| Medication History | | ✓ |  |  |
| Current medications & Confirm washout/prohibited medicines | | ✓ | ✓ |  |
| Physical Examination | | ✓ |  |  |
| Height | | ✓ |  |  |
| Weight | | ✓ |  |  |
| Body Temperature | | ✓ |  |  |
| Vital Signs (BP, HR) | | ✓ |  |  |
| WISC-IV/WAIS-IV | | ✓ |  |  |
| ADOS-2 (ASD group) | | ✓ |  |  |
| SCQ (TDC group) | | ✓ |  |  |
| K-SADS | | ✓ |  |  |
| SRS-2 | | ✓ |  |  |
| EQ-40 | | ✓ |  |  |
| CBCL | | ✓ |  |  |
| TRF | | ✓ |  |  |
| YSR | | ✓ |  |  |
| NCBRF-TIQ | | ✓ |  |  |
| MOAS | | ✓ |  |  |
| CPRS | | ✓ |  |  |
| ICU | | ✓ |  |  |
| BRIEF | | ✓ |  |  |
| C-GAS | | ✓ |  |  |
| CGI | | ✓ |  |  |
| E4 wristband (HR and skin conduct) | |  | ✓ | ✓ |
| Salivar Cortisol  Level | |  |  | ✓ |
| MOT | |  | ✓ |  |
| IED | |  | ✓ |  |
| FEERT | |  | ✓ |  |
| DD | |  | ✓ |  |
| MJ | |  | ✓ |  |
| PD | |  | ✓ |  |
| RVIP | |  |  | ✓ |
| DMS | |  |  | ✓ |
| PRT | |  |  | ✓ |
| NCGT | |  |  | ✓ |
| FA go no go | |  |  | ✓ |
| RLT | |  |  | ✓ |
| ToM | |  |  | ✓ |
| UG | |  |  | ✓ |
