# Supplementary material for: Social and executive functioning in individuals with autism spectrum disorder without intellectual disability: The case–control study protocol of the CNeSA study
Source: Front Child Adolesc Psychiatry. 2023 Apr 21;2:1149244. doi: 10.3389/frcha.2023.1149244 (PMC11731623; doi:10.3389/frcha.2023.1149244)
Supplement: Supplementary file 2 [file Table2.docx]

**Supplementary Material Table (S2): clinical assessment**

| **Category** | **Clinical assessment tools** | **Outcomes** |
| --- | --- | --- |
| Intellectual level | WISC-IV/WAIS-IV | Intelligence level |
| Autism symptoms | Autism Diagnostic Observational Scale-Second Version (ADOS-2) and Autism Diagnostic Interview-Revised (ADI-R) | Autistic symptoms severity |
|  | Social Communication Questionnaire (SCQ) | Communication skills and social functioning in children who may have autism spectrum disorders |
|  | Social Responsiveness Scale-2 | Social impairment and repetitive and restrictive behaviour associated with Autism Spectrum Disorder filled out by the parents |
|  | Empathy Quotient-40 (EQ-40)  Version for children (5-11), adolescents (12-15) and adults (>16) | Cognitive and Emotional Empathy |
| Internalizing/ Externalizing problems | Child Behavior Checklist (CBCL)  Teacher Report Form (TRF)  Youth Self Report (YSR) | General functioning as well as internalizing and externalizing problems fill out by a teacher, the children, the parents |
| Problem behaviours | Kiddie-SADS-PL | Assessment of psychopathology based on DSM-IV categories |
|  | Conner’s Parent Rating Scale-Revised Short Form (CPRS-RS) | Abbreviated version of the factor-derived subscales that assess a cross-section of ADHD-related symptoms and problem behaviours. |
|  | The Nisonger Child Behavior Rating Form (NCBRF-TIQ) | Social competence, disruptive behaviour and ADHD problems |
|  | Modified Overt Aggression Scale (MOAS) | Assessment of aggression: verbal, against property, physical and auto-aggression. |
|  | Inventory of Callous Unemotional Traits (ICU) | Callous Unemotional Traits |
|  | C-GAS | Global evaluation of severity of symptoms |
|  | CGI | Global evaluation of severity of symptoms |
| Executive Functions questionnaire | BRIEF | Assessment of executive function behaviours at home and school |
