# Supplementary material for: Social and executive functioning in individuals with autism spectrum disorder without intellectual disability: The case–control study protocol of the CNeSA study
Source: Front Child Adolesc Psychiatry. 2023 Apr 21;2:1149244. doi: 10.3389/frcha.2023.1149244 (PMC11731623; doi:10.3389/frcha.2023.1149244)
Supplement: Supplementary file 3 [file Datasheet1.docx]

**Supplementary Materials (SM1): Description of screening and clinical instruments**

- **WISC-IV (Wechsler Intelligence Scale for Children – Fourth Edition)** (Wechsler D, 2003) or **WAIS-IV (Wechsler Adult Intelligence Scale-IV)** (Wechsler D, 2008) depending on age of the participant is used to investigate intellectual functioning (if administered within two years before the screening visit, data already collected are used).

- **Kiddie Schedule for Affective Disorders and Schizophrenia - Present and Lifetime Version (Kiddie-Sads-PL)** (Kaufman J, 1997) a semi-structured interview assessing psychopathology based on DSM-IV categories used to diagnose of disorders in comorbidity administered only to parent/guardian.

- **Autism Diagnostic Interview-Revised (ADI-R)** (Lord C, 1994) a semi-structured, investigator-based interview and the **Autism Observational Scale - Second Edition (ADOS-2)** (Lord C, 2012) a semi-structured, standardized assessment of communication, social interaction, play and imagination.

- Parents of the participants included in TDC group completed the **Social Communication Questionnaire** **(SCQ)** (Rutter M, 2003) a brief parent-rated instrument designed to evaluate communication skills and social functioning in children who may have autism or autism spectrum disorders. A score of 10 is used as cut-off for excluding autism in the participant.

**Clinician forms:**

- **Modified Overt Aggression Scale (MOAS)** (Kay SR, 1998): a short rating scale that assesses verbal and physical aggression, aggression against property and auto-aggression. This instrument is administered by the clinical to the parent/caregiver.

- **The Nisonger Child Behaviour Rating Form (NCBR-TIQ)** parent version (Aman M, 2008): a 66-item measure administered by the clinician to the parents/caregiver and used to evaluate child and adolescent behaviour in children with Disruptive Behaviour Disorder

- **Clinical Global Impression-Severity (CGI-S)** (Guy W, 1976) a one-item scale based on the clinician’s assessment of the severity of symptoms in relation to the clinician’s total experience. Severity is rated on a 7-point scale (1=normal, not at all ill; 7= among the most extremely ill subjects).

- **Children’s Global Assessment Scales (C-GAS)** (Shaffer D, 1983): a one-dimensional clinician rating measuring the illness severity and the level of impairment in the social, family, academic and psychiatric functioning. Scores on the measure range from 1 (most impaired, persistent risk to hurt) to 100 (healthiest; no symptoms).

**Parents and patients forms:**

- **Social Responsiveness Scale – Second Edition (SRS-2)** (Constantino JN, 2012) school-age version, a questionnaire filled by the parent or caregiver aims to identify the presence and the level of severity of social impairment related to autistic symptoms.

- **Child Behavior Checklist (CBCL), Teacher Report Form (TRF) and Youth Self Report (YSR)** (Achenbach TM, 1991) standardized questionnaires belonging to Achenbach System of Empirically Based Assessment (ASEBA) used to measure the internalizing and externalizing problems in different contexts.

- **Conner’s Parent Rating Scale-Revised (CPRS-RS)** (Conners KC, 2007), short version filled by parent or caregiver assessing symptoms and problem behaviours related to ADHD in the past month.

- **Behavior Rating Inventory of Executive Function (BRIEF)** (Gioia G, 2000), a questionnaire filled by parents designed to assess the executive functions of children and adolescent between 5 and 18 years old on everyday life.

- **Empathy Quotient** **(EQ-40)** (Baron-Cohen S, 2004) a parent questionnaire for evaluating the cognitive and affective empathy. Different versions of the questionnaire are used depending on the age of participant.

- **Inventory of Callous Unemotional Traits (ICU)** (Essau CA, 2006): a 24 items questionnaire administered to parents designed to provide a comprehensive assessment of CU traits and including three subscales (Callousness, Uncaring and Unemotional).

**Supplementary Materials (SM2): Neuropsychological Assessment**

**Visit 0a**

**Motor screening (MOT)** **CANTAB battery**

MOT is a simple reaction time screening test with the aim to make the subject familiar with the test material and to evaluate the presence of any limitations in the use of the device (vision or hearing problems, motors, etc.).

**Intra Extra Dimensional Set Shifting (IED) CANTAB battery**

IED is a test requiring attentional set formation, rule maintenance and set-shifting abilities.

Outcome measures: this task comprises 18 outcome measures that can be grouped in accuracy (total errors) and shifting abilities measures (number of trials and stages completed).

**Faces and Eyes Emotional Recognition Task (FEERT) Emoticom battery**

FEERT measures the ability to process and identify the four basic emotions (happiness, sadness, anger and fear) in facial and eyes expressions presented with different intensities.

Outcome measures: the accuracy in identifying each emotion evaluated computing the total number of errors through each emotion. The affective bias measure calculated subtracting the accuracy for sad expressions from the accuracy for happy expressions. The latency of the response expressed in reaction times for each emotion.

**Delay Discounting (DD) Emoticom battery**

DD task provides the measures of inhibition/impulsivity and delay aversion based on the rate of discount across delays or degrees of uncertainty.

Outcome measures: Area under the curve (AUC) and k calculated from indifference points.

**Moral Judgment (MJ) Emoticom battery**

MJ task assesses the normative emotional reactions in front of scenarios presented by cartoon figures depicting moral situations. The participant rates the intensity of some emotions (guilt, shame, annoyance, and good/bad) felt across different conditions in which he identify himself with the victim or victimizer, combined with a situation in which an intentional or unintentional harm is acted.

Outcome measures: score on intensity of the emotions across all four combined conditions.

**Prisoner Dilemma (PD) Emoticom battery**

PD task evaluates the cooperation ability towards opponents who use different cooperative behaviours (progressively unfair) and the proportion of profit gathered compared to the opponent (more, less, equal). The opponent strategies are named: aggressive (tit for tat but starts with steal), tit for two tats (starts with split and reproduces the player behaviour after two times he implements), and cooperative (always splits).

Outcome measure: cooperative behaviour as rate of split across all situations and response latency.

**Visit 0b**

**Rapid Visual Processing (RVP) CANTAB battery**

RVP is a test measuring the visual sustained attention.

Outcome measures: accuracy (errors and correct responses), latency (mean reaction times) and target sensibility.

**Delay Matching to Sample (DMtS) CANTAB battery**

DMtS measures both the visual matching and the visual recognition memory in a forced choice task. In the recognition trials the delay between the target stimulus and the choice patterns changes to have three conditions of delay (0, 4, 12 sec. of delay).

Outcome measures: this test provides several measures related to response times, accuracy (proportion of correct responses) in all conditions.

**Progressive Ratio Task (PRT) Emoticom battery**

PRT measures participants’ motivational ‘breakpoint’ and the incentive motivation of how much effort an individual is prepared to exert to gain a reward.

Outcome measures: number of completed trials, post reinforcement pause (average time taken to initiate the next trial following a reward), the running rate (time taken to complete the block of trials).

**The New Cambridge Gambling Task (NCGT) Emoticom battery**

NCGT evaluates how subjects use different type of information (e.g. probability, reward or punishment condition) in order to guide economic decision-making, the risk-taking behaviour and the sensitivity to punishment and reward pit side a learning context.

Outcome measures: the output measures evaluate the quality of decision making and the decision-making behaviour, proportion of bet in win and loss conditions and overall.

**Face Affective Go-no Go Task (FAGNG) Emoticom battery**

The FAGNG task is used to investigate the attentional emotional bias that is the information processing biases for positive and negative facial expression.

Outcome measures: reaction times (RT) for the correct responses in each condition, the affective bias and the accuracy expressed computing the proportion of Hits, Misses, Correct Rejections and False Alarms.

**The Reinforcement Learning Task (RLT) Emoticom battery**

The RLT is a task evaluating the ability to learn, apply effort and make decision driven by incentives. It measures the sensitivity to reward and punishment through use of operant conditioning principles.

Outcome measures: reaction times response (RT) and learning rate.

**Ultimatum Game (UG) Emoticom battery**

UG assesses the fairness sensitivity in response to a progressively more unfair opponent’s behaviour and the punishment tendency.

Outcome measures: the fairness sensitivity obtained with the proportion of acceptance behaviour in response to each level of opponent offer (90%, 80% 70%, 60%, 50%). The punishment tendency is obtained with the average offer proportion of the participant. Risk adjustment is calculated also.

**Theory of Mind (ToM) Emoticom battery**

ToM task investigates the information type preference to resolve socially ambiguous situations and the valence of the interpretation.

Outcome measures: proportion of feelings, thought and facts stimuli chosen by the participant, outcome choice (negative, positive, or neutral) and outcome choice confidence.
